# Reduced functional connectivity of the right dorsolateral prefrontal cortex at rest in obsessive–compulsive disorder

**DOI:** 10.1002/brb3.3333

**Published:** 2024-01-11

**Authors:** Zhaoxi Zhong, Yangpan Ou, Yunhui Chen, Ping Li, Han Shi, Dan Lv, Cuicui Jia, Tinghuizi Shang, Lei Sun, Ru Yang, Xiaoping Wang, Wenbin Guo, Luxian Lv

**Affiliations:** ^1^ Henan Key Laboratory of Biological Psychiatry The Second Affiliated Hospital of Xinxiang Medical University Xinxiang Henan China; ^2^ National Clinical Research Center for Mental Disorders and Department of Psychiatry The Second Xiangya Hospital of Central South University Changsha Hunan China; ^3^ Department of Psychiatry Qiqihar Medical University Qiqihar Heilongjiang China; ^4^ Department of Radiology The Second Xiangya Hospital of Central South University Changsha Hunan China

**Keywords:** functional connectivity, functional magnetic resonance imaging, obsessive–compulsive disorder, resting‐state, right dorsolateral prefrontal cortex

## Abstract

**Background:**

Neuroimaging studies have revealed the role of the right dorsolateral prefrontal cortex (DLPFC) in the neurobiological mechanism of obsessive–compulsive disorder (OCD). However, only a few studies have examined the functional connectivity (FC) pattern of the right DLPFC at rest in OCD.

**Objective:**

The aim of this research is to examine the FC patterns of the right DLPFC at rest in OCD.

**Methods:**

Twenty‐eight medication‐free patients with OCD and 20 healthy controls underwent resting‐state functional magnetic resonance imaging. Seed‐based FC and support vector machine (SVM) were used to analyze the imaging data.

**Results:**

The patients with OCD showed reduced FC values in the right middle temporal gyrus (MTG), right superior temporal gyrus, right ventral anterior cingulate cortex (vACC), and left Crus II. No brain regions showed a remarkable difference in FC values in patients with OCD after 8 weeks of medication treatment. The reduced right DLPFC–right MTG and right DLPFC–right vACC connectivities were correlated with the clinical symptoms of OCD. SVM results showed that reduced right DLPFC–right MTG connectivity at rest could predict the therapeutic response to OCD medication.

**Conclusions:**

The findings highlight the important role of the right DLPFC in the pathophysiological mechanism of OCD.

## Graphical abstract

Only a few studies have examined the functional connectivity (FC) pattern of the right DLPFC at rest in OCD. We examined the functional connectivity (FC) patterns of the right DLPFC at rest in OCD. Patients showed abnormal FC values of the right DLPFC and some brain regions. Support vector machine (SVM) results showed that reduced right DLPFC–right MTG connectivity at rest could predict the therapeutic response to OCD medication. The findings highlight the important role of the right DLPFC in the pathophysiological mechanism of OCD.

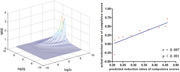

## 1 INTRODUCTION

Obsessive−compulsive disorder (OCD) is a chronic disabling mental disorder with 2.3% lifetime prevalence (Ruscio et al., 2010). The main clinical symptoms of obsessions and compulsions severely affect the personal life and social function of patients with OCD (Ruscio et al., 2010). Cortico–striato–thalamo–cortical (CSTC) circuit abnormalities and structural and functional brain changes are involved in the neurobiological mechanism of OCD (Ahmari et al., 2013; Baldermann et al., 2019).

The role of the dorsolateral prefrontal cortex (DLPFC), a crucial brain region in the CSTC circuit, in the pathophysiology of OCD has elicited much attention. The meta‐analysis of voxel‐based morphometry studies found that patients with OCD had reduced gray matter volume in bilateral DLPFC compared to controls (Norman et al., 2016). Several studies tested repetitive transcranial magnetic stimulation (rTMS) of the left or right DLPFC as a treatment for OCD. The rationale is that DLPFC is involved in cognitive control and inhibitory processes that may be dysfunctional in OCD (Zhou et al., 2017). Many neuroimaging studies have discovered structural and functional abnormalities in DLPFC, which is involved in the impairment of cognitive flexibility and executive planning in OCD (Baldermann et al., 2019; Chen et al., 2016; Menzies et al., 2008; Park et al., 2016; Peng et al., 2012; Tomasino & Fabbro, 2016; Yang et al., 2015, 2019). Compared with the left DLPFC, the right DLPFC is more sensitive to OCD (Park et al., 2016). For example, a meta‐analytics study (Peng et al., 2012) found that reduced gray matter volume in the right DLPFC is correlated with clinical symptoms of OCD (Alvarenga et al., 2012). The brain metabolism of glutamate/glutamine and myo‐inositol is abnormal in the right DLPFC in patients with OCD (Park et al., 2016). Moreover, regional homogeneity (ReHo) in the right DLPFC is higher in patients with OCD and their unaffected siblings, suggesting that increased ReHo in the right DLPFC might be an endophenotype for OCD (Yang, Luo et al., 2019). Furthermore, the stimulation of the right DLPFC through rTMS targeting is more effective than left DLPFC stimulation in decreasing OCD symptoms (Sachdev et al., 2001). In deep brain stimulation (DBS) for OCD, the connectivity of the ventral internal capsule to the right DLPFC is correlated with the clinical outcome (Baldermann et al., 2019), and activation in the right DLPFC increases after 8 weeks of focused attention‐based mindfulness meditation (MT) (Tomasino & Fabbro, 2016). Therefore, the right DLPFC plays a crucial role in the pathophysiological mechanism of OCD and may be related to the treatment outcome for OCD.

The contribution of the right DLPFC to the pathophysiological mechanism of OCD may be demonstrated by abnormalities in other brain regions because of reciprocal functional connections between the right DLPFC and other brain regions (Ding et al., 2019). However, to our best knowledge, only a few studies have examined the functional connectivity (FC) pattern of the right DLPFC at rest in OCD.

In the current study, we explored the FC pattern of the right DLPFC at rest in medication‐free patients with OCD by using resting‐state functional magnetic resonance imaging (RS‐fMRI). The objectives of the study are to (1) examine the FC pattern of the right DLPFC at rest in OCD, (2) explore the correlations between abnormal FC of the right DLPFC and clinical variables of OCD, (3) investigate the changes in FC in the right DLPFC before and after medication treatment, and (4) examine whether the altered FC patterns of the right DLPFC can be used to predict the therapeutic response in OCD. We hypothesized that OCD manifests altered FC patterns of the right DLPFC at rest and is correlated to the clinical characteristics of OCD, which change after medication treatment. We also hypothesized that these alterations in the right DLPFC connectivity can be used to predict the therapeutic response of patients with OCD.

## 2 MATERIALS AND METHODS

### 2.1 Participants

Twenty‐eight right‐handed patients with OCD were recruited from the Department of Psychiatry, Second Affiliated Hospital of Xinxiang Medical University, China. Twenty‐two right‐handed healthy controls (HCs) were recruited from the community. All participants were 18–60 years old. The patients were diagnosed with OCD on the basis of the structured clinical interview for DSM‐IV Axis I Disorder–Patient Edition (SCID‐I/P) (First et al., 1996). The Yale–Brown Obsessive–compulsive scale (Y‐BOCS) was used to assess OCD severity, and the patients scored higher than 16 in Y‐BOCS. All patients were medication‐free for at least 4 weeks at the baseline. The HCs were examined using SCID‐I/NP (non‐patient version). Participants with other Axis I disorders, such as schizophrenia, depression, bipolar disorder, substance‐induced mood disorder, anxiety disorder, history of seizures, cerebrovascular disease, and significant head injury, were excluded. HCs with a family history of psychiatric disorders were also excluded.

Eleven patients from the total sample were treated with selective serotonin reuptake inhibitors (i.e., sertraline, fluvoxamine, paroxetine, fluoxetine, and citalopram) for 8 weeks. These 11 patients with OCD were assessed and scanned at 2 time points (baseline and after 8 weeks of medication treatment). The reliable change index (RCI) was used to define responders and nonresponders in terms of medication. RCI was defined as a score change (baseline vs. week 8) in Y‐BOCS divided by the standard error of the difference (Costa & Paula, 2015). When RCI was equal to or more than 1.96, the patients were regarded as responders (Kneebone et al., 1998). In accordance with this criterion, 11 patients with OCD were considered responders.

Clinical ratings of OCD symptoms were independently performed by two psychiatrists blinded to subject diagnosis and prior scores. The ratings from the two clinicians were then averaged for each subject.

### 2.2 Image data acquisition and preprocessing

Imaging was performed using a 3.0‐Tesla Siemens MRI scanner (Siemens) in the Second Affiliated Hospital of Xinxiang Medical University, China. The subjects were required to stay awake, keep their eyes closed, and remain motionless (especially the head). RS‐fMRI data were acquired with an echo‐planar imaging sequence. The parameters were as follows: 33 axial slices, TE = 30 ms, TR = 2000 ms, thickness/gap = 4.0 mm/0.8 mm, FA = 90°, in‐plane resolution = 64 × 64, and FOV = 220 × 220 mm^2^.

The Data Processing & Analysis for Brain Imaging software was used to preprocess the imaging data. Data processing was performed in reference to our previously published stuvvdies (Chen et al., 2019; Cui et al., 2020), and the details are provided in Supporting Information.

### 2.3 Functional connectivity analysis

The seed‐based FC approach was used to explore the FC patterns of the right DLPFC in all the participants. The right DLPFC seed, including Brodmann's area [BA] 9/46, was conceptualized as previously described. The RS‐fMRI data analysis toolkit (REST) was used to create the region‐of‐interest mask (Figure S1). For each subject, correlation maps were built by performing Pearson correlation analyses between the right DLPFC and other brain voxels of the entire brain. The FC values were transformed into a *Z*‐score map via Fisher‐*Z* transformation to improve normality.

### 2.4 Support vector machine analysis

Support vector machine (SVM) was applied with the LIBSVM software (http://www.csie.ntu.edu.tw/cjlin/libsvm/) to determine if the altered right DLPFC connectivity can be used to predict the therapeutic response to OCD medication. A “leave‐one‐out” approach was used to perform SVM.

### 2.5 Statistical analysis

The demographic and clinical characteristics of the two groups were analyzed via two‐sample *t*‐tests or a *χ* ^2^‐test by using SPSS Version 23.0 (SPSS Inc.).

One‐sample *t*‐tests were conducted to identify voxels with significant correlations with the right DLPFC for each group. The threshold level was set to *p* < .05 and corrected by Gaussian random field (GRF). Voxel‐wise two‐sample *t*‐tests were then performed to compare group differences (OCD vs. HCs). The mean framewise displacement and age were used as covariates. Voxel‐wise paired *t*‐tests were conducted to compare FC changes between 8 weeks of medication treatment and the baseline. The threshold level was *p* < .05 (cluster‐based GRF correction (voxel significance *p* < .001; cluster significance *p* < .05, two‐tailed)).

Pearson correlation analyses were performed between the mean *z* values from brain clusters with altered FC values and clinical characteristics in the treatment sample (11 patients with OCD) at the baseline. The threshold level was *p* < .05 (Bonferroni corrected).

### 2.6 Ethics

The study procedures were explained, and a written informed consent form was signed by all participants. The study was approved by the Research Ethics Committee of the Second Affiliated Hospital of Xinxiang Medical University, China.

## 3 RESULTS

### 3.1 Characteristics of the participants

Two HCs were excluded from further analysis due to excessive head motion. First, a normality test “One Sample Kolmogorov Smirnov Test” was conducted on the age of the case group and the control group, following a normal distribution. In the total sample, no significant differences were observed in terms of age, gender, and education between the OCD and HC groups. However, the patients with OCD had high Y‐BOCS total scores, Y‐BOCS obsessive thinking subscale scores, and Y‐BOCS compulsive behavior subscale scores (Table 1).

**TABLE 1:** Demographic and clinical characteristics of the total participants at baseline.

	OCD (*n* = 28) Baseline	HCs (*n* = 20) Baseline		*χ^2^/t*	*p*
Age (years)	23.25 ± 6.07	25.46 ± 8.85		−.97	.08
Sex (male/female)	13/15	10/10		.81	.06
Education (years)	10.85 ± 2.50	11.43 ± 2.92		−.72	.49
Illness duration (months)	36.79 ± 45.68	–		–	–
Y‐BOCS total score	27.71 ± 8.89	–		–	–
Y‐BOCS obsessive thinking	13.96 ± 4.61	–		–	–
Y‐BOCS compulsive behavior	13.75 ± 4.62	–		–	–

*Note*: Data are presented as mean ± standard deviation or number. The variable such as gender was analyzed using a chi‐square test, whereas other variables were analyzed using two‐sample *t‐*tests.

Abbreviations: HCs, healthy controls; OCD, obsessive−compulsive disorder; Y‐BOCS, Yale–Brown Obsessive−compulsive scale.

In the treatment sample, OCD‐related symptoms were significantly reduced after 8 weeks of medication treatment (all *p* < .05) (Table 2). The subset (Y‐BOCS total score, Y‐BOCS obsessive thinking, Y‐BOCS compulsive behavior) of participants who underwent 8 weeks treatment did not have statistically different YBOCS subscale scores relative to the subset who did not undergo the 8 weeks treatment (*t* = .88, *p* = .381; *t* = .72, *p* = .456; *t* = .56, *p* = .598).

**TABLE 2:** Demographic and clinical characteristics of obsessive–compulsive disorder (OCD) before and after medication treatment.

	OCD (*n* = 11) at baseline	OCD (*n* = 11) after treatment	*t*	*p*
Y‐BOCS total score	30.45 ± 10.88	18.09 ± 7.35	3.12	.005
Y‐BOCS obsessive thinking	15.55 ± 5.85	9.09 ± 3.36	3.17	.005
Y‐BOCS compulsive behavior	14.91 ± 5.47	9.00 ± 4.54	2.76	.012

*Note*: The variables were analyzed using paired‐sample *t*‐tests; the *p* values indicate the comparisons between baseline and 8 weeks of medication treatment in OCD.

Abbreviation: Y‐BOCS, Yale–Brown Obsessive−compulsive scale.

### 3.2 One‐sample *t*‐test on seed‐based FC

In the HC group, the frontal, temporal, occipital, and parietal cortex and the cerebellum showed positive FC patterns with the right DLPFC. The patients’ group presented similar FC patterns with the right DLPFC as the HCs (Figure S2). No similar findings for the left DLPFC were showed in the two groups.

### 3.3 Two‐sample *t*‐test on seed‐based FC

**FIGURE 1:**
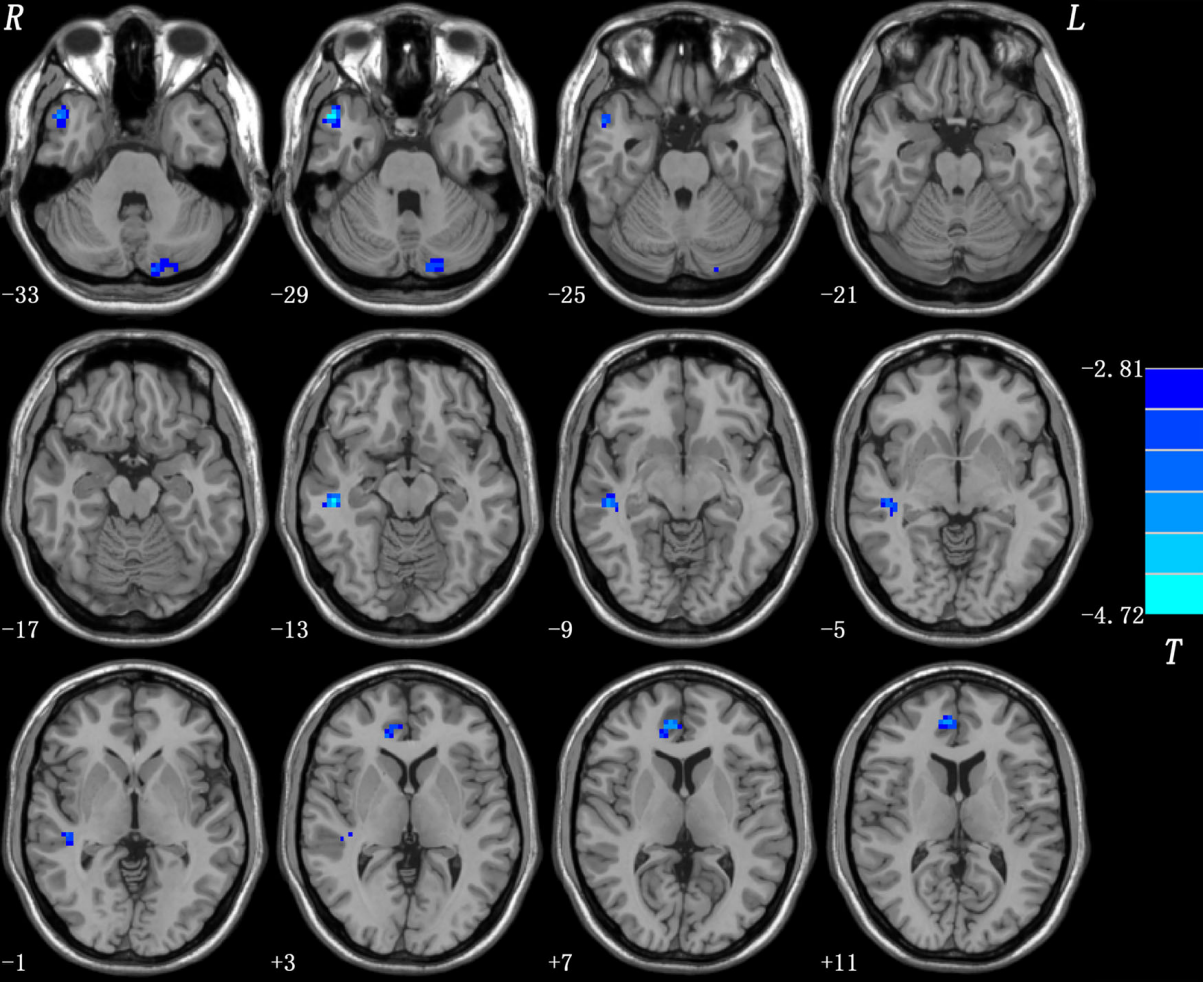
Brain regions showing group differences of the right dorsolateral prefrontal cortex (DLPFC) functional connectivity at baseline in obsessive–compulsive disorder (OCD). The threshold was set at *p* < .05 corrected by Gaussian random field (GRF). Blue denotes reduced functional connectivity in OCD. Color bar indicates the *t* values from two‐sample *t*‐tests. L, left side; R, right side.

Compared with the HCs, the OCD patients had significantly reduced right DLPFC connectivity in the right middle temporal gyrus (MTG), right superior temporal gyrus (STG), right ventral anterior cingulate cortex (vACC), and left Crus II (Table 3 and Figure 1).

**TABLE 3:** Brain regions with abnormal functional connectivity with the right dorsolateral prefrontal cortex (DLPFC) at baseline in obsessive–compulsive disorder (OCD).

Cluster location	Peak (MNI)			Number of voxels	*t* Value
	*x*	*y*	*z*		
Right middle temporal gyrus	51	12	−30	53	−4.7208
Right superior temporal gyrus/middle temporal gyrus	51	−24	−12	57	−4.4555
Right ventral anterior cingulate cortex	9	48	9	40	−4.6801
Left Crus II	−12	−90	−33	41	−3.5558

Abbreviation: MNI, Montreal Neurological Institute.

Compared with the situation at the baseline, no brain regions showed a remarkable difference in right DLPFC connectivity in the patients with OCD after 8 weeks of medication treatment.

### 3.4 Correlations between FC values and clinical characteristics in OCD

**FIGURE 2:**
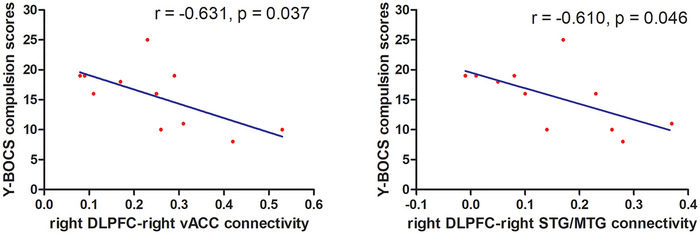
Correlations between functional connectivity and clinical variables in obsessive–compulsive disorder (OCD). The significance level was Bonferroni corrected at *p* < .05. DLPFC, dorsolateral prefrontal cortex; vACC, ventral anterior cingulate cortex; Y‐BOCS, Yale–Brown Obsessive–compulsive scale.

In the all patients, no significantly correlations were observed between YBOCS scores and FC at the baseline. In the treatment sample, significantly negative correlations were observed between the right DLPFC–right MTG connectivity and the Y‐BOCS compulsion behavior scores (*r* = −.610, *p* = .046; Bonferroni corrected) and between the right DLPFC–right vACC connectivity and the Y‐BOCS compulsion behavior scores (*r* = −.631, *p* = .037; Bonferroni corrected) at the baseline (Figure 2). No significant relationships were found between changes in Y‐BOCS scores and changes in FC values in patients with OCD before and after 8 weeks of medication treatment.

### 3.5 SVM results

**FIGURE 3:**
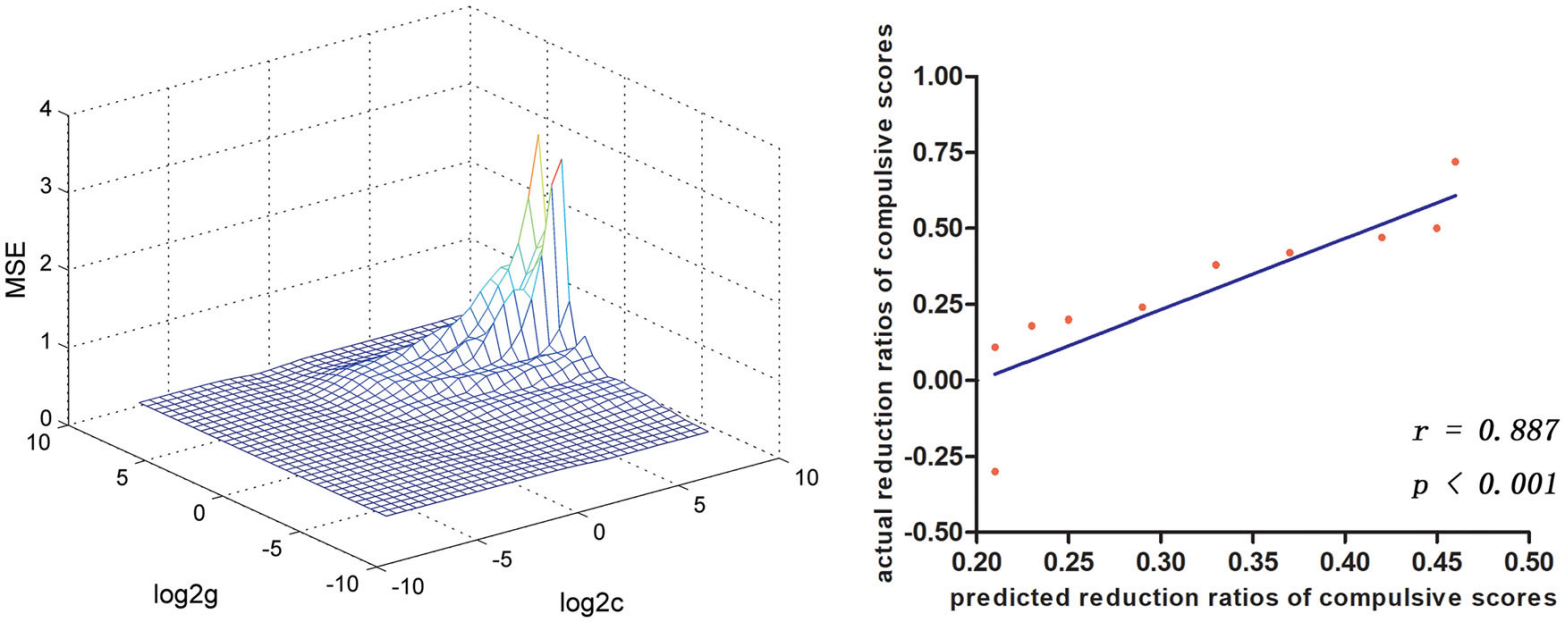
Support vector machine (SVM) results show that reduced right DLPFC–right middle temporal gyrus (MTG) connectivity predict the therapeutic response to obsessive–compulsive disorder (OCD) medication. Left: SVM parameters selection results (3D visualization); right: a positive correlation between actual and predicted reduction ratios of the compulsive scores of individual OCD (*r* = .887, *p* < .001). DLPFC, dorsolateral prefrontal cortex.

SVM analysis was conducted using the FC values (right vACC and right MTG) that revealed negative correlations with clinical variables in the treatment sample. The actual versus predicted reduction ratios of the compulsive scores of right vACC was *r* = .189, *p* = .579; the actual versus predicted reduction ratios of the compulsive scores of right MTG was *r* = .887, *p* = .001 (Figure 3). The SVM results show that reduced right DLPFC–right MTG connectivity can predict the therapeutic response to OCD medication.

## 4 DISCUSSION

This study explored the FC patterns of the right DLPFC at rest in OCD. Consistent with our hypothesis, the patients with OCD displayed reduced FC patterns of the right DLPFC within the CSTC circuitry (i.e., right vACC) and outside the CSTC circuitry (i.e., right MTG, right STG, and left Crus II). The right DLPFC–right MTG and right DLPFC–right vACC connectivities were negatively correlated with the compulsion behavior scores in OCD. Furthermore, reduced right DLPFC–right MTG connectivity could be used to predict the therapeutic response to OCD medication.

The current study found reduced right DLPFC–right MTG and right DLPFC–right STG connectivities at rest in OCD. Previous studies have reported reduced regional activities in MTG and STG at rest that are correlated with insight level and anhedonia severity in OCD (Fan et al., 2017; Xia et al., 2019). These findings reveal the role of MTG and STG dysfunction in the pathophysiology of OCD; MTG and STG are considered gene‐related endophenotypes of OCD (Hou et al., 2014). Reduced right DLPFC–right MTG and right DLPFC–right STG connectivities suggest disruptive coordinated functions within and outside the CSTC circuitry at rest in OCD.

Reduced right DLPFC–right vACC connectivity at rest in OCD was discovered in the present study. In our previous published study, we reported a reduced ReHo value in vACC in a different sample of OCD (Ping et al., 2013). Reduced right DLPFC–right vACC connectivity at rest implies that the right DLPFC contributing to the pathophysiology of OCD may manifest reduced connectivity with the right vACC, which may cause destructive imbalance between the dorsal and ventral CSTC circuitry at rest in OCD (Menzies et al., 2008).

Studies on neuroimaging have linked cerebellum abnormalities with neuropsychology deficits in patients with OCD to increased gray matter volume, reduced amplitude of low‐frequency fluctuations (ALFF), and increased ReHo at rest (Hu et al., 2017; Meng et al., 2018; Ping et al., 2013; Qiu et al., 2017). Moreover, patients with OCD show increased cerebellar–DMN connectivity and reduced cerebellar–cerebral connectivity in emotion processing networks (Xu et al., 2019). In the current study, the reduced right DLPFC–left Crus II connectivity implies reduced cerebellar–cerebral connectivity in the executive network, which may be correlated with excessive attention to unrelated external stimulation and excessive anxiety in OCD (Xu et al., 2019).

This study also examined the changes in the right DLPFC connectivity after OCD medication treatment. After 8 weeks of medication treatment, the clinical symptoms of OCD were significantly reduced; however, the FC patterns of the right DLPFC did not change. We infer that the treatment was too short to produce changes in the FC pattern of the right DLPFC in the relatively small OCD sample; these changes could be tested after long‐term medication treatment in a large OCD sample.

In the treatment sample, we found negative relationships between right DLPFC–right MTG and right DLPFC–right vACC connectivities and the Y‐BOCS compulsion behavior scores at the baseline. MTG is involved in cognitive processes (i.e., semantic memory and language) (Fox et al., 2006), and vACC is involved in assessing and regulating emotional information and responses (Bush et al., 2000). The reduced right DLPFC–right MTG and right DLPFC–right vACC connectivities at rest may reflect underlying problems in the integration of cognitive information (i.e., response inhibition, planning, and memory) and cognitive inflexibility in OCD; these problems may have contributed to the negative relationship between reduced right DLPFC–right MTG and right DLPFC–right vACC connectivities and compulsive symptoms of OCD found in the current study.

The SVM analysis revealed that the reduced right DLPFC–right MTG connectivity can predict the therapeutic response to OCD medication. This result means that a small reduction in right DLPFC–right MTG connectivity at rest equates to a good medication treatment effect on OCD. Previous studies have revealed the relationship between the right DLPFC and the therapeutic effect of brain stimulation treatments (i.e., rTMS and DBS) and psychotherapy (i.e., MT) (Baldermann et al., 2019; Sachdev et al., 2001; Tomasino & Fabbro, 2016). Moreover, the fractional ALFF value in the right MTG can be used to distinguish people with OCD from HCs (Yang et al., 2019). On the basis of the current and previous findings, we infer that the right DLPFC–right MTG connectivity at rest can be used as a potential neuroimaging biomarker to predict the therapeutic effect of OCD medication.

## 5 LIMITATIONS

Several limitations of this work must be considered. First, the size of the OCD sample was small. Second, cognitive and behavioral information were not collected. As a result, we could not explore the correlation between cognitive dysfunction and altered FC patterns in OCD. Third, the time of medication treatment was too short to explore FC changes. Therefore, the changes in reduced right DLPFC connectivity in relation to the time of medication treatment require further longitudinal research.

## 6 CONCLUSION

In summary, the present study revealed reduced right DLPFC FC patterns within and outside the CSTC circuitry at rest in OCD. Reduced right DLPFC–right MTG connectivity at rest can be used as a potential neuroimaging biomarker to predict the therapeutic effects of OCD medication. The results highlight the role of the right DLPFC in the pathophysiological mechanism of OCD, and this role may be manifested by reduced connectivities with other brain regions located within and outside the CSTC circuitry.

## PUBLISHER'S NOTE

Springer Nature remains neutral with regard to jurisdictional claims in published maps and institutional affiliations.

## OPEN ACCESS

This article is distributed under the terms of the Creative Commons Attribution 4.0 International License (http://creativecommons.org/licenses/by/4.0/), which permits unrestricted use, distribution, and reproduction in any medium, provided you give appropriate credit to the original author(s) and the source, provide a link to the Creative Commons license, and indicate if changes were made. The Creative Commons Public Domain Dedication waiver (http://creativecommons.org/publicdomain/zero/1.0/) applies to the data made available in this article, unless otherwise stated.

## ADDITIONAL FILES

The detailed clinical and demographic data for all participants are shown in Tables 1 and 2. The patients with OCD showed reduced FC values in the right middle temporal gyrus (MTG), right superior temporal gyrus, right ventral anterior cingulate cortex (vACC), and left Crus II (Table 3, Figure 1). The reduced right DLPFC–right MTG and right DLPFC–right vACC connectivities were correlated with the clinical symptoms of OCD (Figure 2). SVM results showed that reduced right DLPFC–right MTG connectivity at rest could predict the therapeutic response to OCD medication (Figure 3).

## AUTHOR CONTRIBUTIONS

Z.Z., Y.O., Y.C., X.W., W.G. and L.X.L. contributed to the conception and design of the study and the acquisition, analysis and interpretation of data. H.S., D.L., C.J. and T.S. contributed to the conception and design of the study and the acquisition of data. L.S. and R.Y. contributed to the conception and design of the study and the analysis of data. Z.Z., P.L. and L.X.L. wrote the article, which all other authors reviewed. All authors gave approval for publication.

## CONFLICT OF INTEREST STATEMENT

The authors have no conflicts of interest to declare.

### PEER REVIEW

The peer review history for this article is available at https://publons.com/publon/10.1002/brb3.3333.

## Supporting information

FIGURE S1 Region‐of‐interest mask of the right dorsolateral prefrontal cortex. DLPFC, dorsolateral prefrontal cortex.

FIGURE S2 Brain regions showing functional connectivities with the right DLPFC within OCD group and HC group. The threshold was set at *p* < .05 corrected by GRF. Blue denotes reduced FC values in the patients. Color bar indicates the *t* values from one‐sample *t*‐tests. *L*, left side; *R*, right side; DLPFC, dorsolateral prefrontal cortex; OCD, obsessive–compulsive disorder; HCs, healthy controls.

Supporting Information

## Data Availability

The datasets used and/or analyzed in this study are available from the corresponding author on reasonable request.

## References

[brb33333-bib-0001] Ahmari, S. E. , Spellman, T. , Douglass, N. L. , Kheirbek, M. A. , Simpson, H. B. , Deisseroth, K. , & Hen, R. (2013). Repeated cortico‐striatal stimulation generates persistent OCD‐like behavior. Science, 340(6137), 1234–1239. 10.1126/science.1234733 23744948 PMC3954809

[brb33333-bib-0002] Alvarenga, P. G. , do Rosario, M. C. , Batistuzzo, M. C. , Diniz, J. B. , Shavitt, R. G. , Duran, F. L. , & Hoexter, M. Q. (2012). Obsessive‐compulsive symptom dimensions correlate to specific gray matter volumes in treatment‐naive patients. Journal of Psychiatric Research, 46(12), 1635–1642. 10.1016/j.jpsychires.2012.09.002 23040160

[brb33333-bib-0003] Baldermann, J. C. , Melzer, C. , Zapf, A. , Kohl, S. , Timmermann, L. , Tittgemeyer, M. , & Kuhn, J. (2019). Connectivity profile predictive of effective deep brain stimulation in obsessive‐compulsive disorder. Biological Psychiatry, 85(9), 735–743. 10.1016/j.biopsych.2018.12.019 30777287

[brb33333-bib-0004] Bush, G. , Luu, P. , & Posner, M. I. (2000). Cognitive and emotional influences in anterior cingulate cortex. Trends in Cognitive Sciences, 4(6), 215–222. 10827444 10.1016/s1364-6613(00)01483-2

[brb33333-bib-0005] Chen, Y. , Meng, X. , Hu, Q. , Cui, H. , Ding, Y. , Kang, L. , & Li, P. (2016). Altered resting‐state functional organization within the central executive network in obsessive‐compulsive disorder. Psychiatry and Clinical Neurosciences, 70(10), 448–456. 10.1111/pcn.12419 27377579

[brb33333-bib-0006] Chen, Y. , Ou, Y. , Lv, D. , Yang, R. , Li, S. , Jia, C. , & Li, P. (2019). Altered network homogeneity of the default‐mode network in drug‐naive obsessive‐compulsive disorder. Progress in Neuro‐Psychopharmacology & Biological Psychiatry, 93, 77–83. 10.1016/j.pnpbp.2019.03.008 30905622

[brb33333-bib-0007] Costa, D. D. S. , & Paula, J. J. D. (2015). Usefulness of the reliable change index for psychology and psychiatry in clinical practice: A case report of cognitive‐behavioral therapy. Clinical Neuropsychiatry, 12(5), 135–138.

[brb33333-bib-0008] Cui, G. , Ou, Y. , Chen, Y. , Lv, D. , Jia, C. , Zhong, Z. , & Li, P. (2020). Altered global brain functional connectivity in drug‐naive patients with obsessive‐compulsive disorder. Front Psychiatry, 11, 98. 10.3389/fpsyt.2020.00098 32194450 PMC7062961

[brb33333-bib-0009] Fan, J. , Zhong, M. , Gan, J. , Liu, W. , Niu, C. , Liao, H. , & Zhu, X. (2017). Spontaneous neural activity in the right superior temporal gyrus and left middle temporal gyrus is associated with insight level in obsessive‐compulsive disorder. Journal of Affective Disorders, 207, 203–211. 10.1016/j.jad.2016.08.027 27723545

[brb33333-bib-0010] First, M. B. , Spitzer, R. L. , Gibbon, M. , & Williams Janet, B. W. (1996). Structured clinical interview for DSM‐IV Axis I disorders, clinician version (SCID‐CV). American Psychiatric Press.

[brb33333-bib-0011] Fox, M. D. , Corbetta, M. , Snyder, A. Z. , Vincent, J. L. , & Raichle, M. E. (2006). Spontaneous neuronal activity distinguishes human dorsal and ventral attention systems. Proceedings of the National Academy of Sciences of the United States of America, 103(26), 10046–10051. 10.1073/pnas.0604187103 16788060 PMC1480402

[brb33333-bib-0012] Hou, J. M. , Zhao, M. , Zhang, W. , Song, L. H. , Wu, W. J. , Wang, J. , & Li, H. T. (2014). Resting‐state functional connectivity abnormalities in patients with obsessive‐compulsive disorder and their healthy first‐degree relatives. Journal of Psychiatry & Neuroscience, 39(5), 304–311. 10.1503/Jpn.130220 24866415 PMC4160359

[brb33333-bib-0013] Hu, X. , Du, M. , Chen, L. , Li, L. , Zhou, M. , Zhang, L. , & Gong, Q. (2017). Meta‐analytic investigations of common and distinct grey matter alterations in youths and adults with obsessive‐compulsive disorder. Neuroscience and Biobehavioral Reviews, 78, 91–103. 10.1016/j.neubiorev.2017.04.012 28442404

[brb33333-bib-0014] Kneebone, A. C. , Andrew, M. J. , Baker, R. A. , & Knight, J. L. (1998). Neuropsychologic changes after coronary artery bypass grafting: Use of reliable change indices. Annals of Thoracic Surgery, 65(5), 1320–1325. 9594860 10.1016/s0003-4975(98)00158-1

[brb33333-bib-0015] Meng, Z. , Zhang, Z. , Fan, Q. , & Li, Y. (2018). Altered fractional amplitude of low frequency fluctuations in unmedicated female patients with obsessive‐compulsive disorder. Annual International Conference of the IEEE Engineering in Medicine and Biology Society. IEEE Engineering in Medicine and Biology Society. Annual Conference, 2018, 1144–1147. 10.1109/EMBC.2018.8512490 30440592

[brb33333-bib-0016] Menzies, L. , Chamberlain, S. R. , Laird, A. R. , Thelen, S. M. , Sahakian, B. J. , & Bullmore, E. T. (2008). Integrating evidence from neuroimaging and neuropsychological studies of obsessive‐compulsive disorder: The orbitofronto‐striatal model revisited. Neuroscience and Biobehavioral Reviews, 32(3), 525–549. 10.1016/j.neubiorev.2007.09.005 18061263 PMC2889493

[brb33333-bib-0017] Norman, L. J. , Steve, C. C. , Hart, L. H. , Joaquim, D. M. , & Rubia, R. K. (2016). Structural and functional brain abnormalities in attention‐deficit/hyperactivity disorder and obsessive‐compulsive disorder: A comparative meta‐analysis. JAMA Psychiatry, 73(8), 815–825. 27276220 10.1001/jamapsychiatry.2016.0700

[brb33333-bib-0018] Park, S. E. , Choi, N. G. , & Jeong, G. W. (2016). Metabolic abnormality in the right dorsolateral prefrontal cortex in patients with obsessive‐compulsive disorder: Proton magnetic resonance spectroscopy. Acta Neuropsychiatrica, 29(3), 164–169. 27748207 10.1017/neu.2016.48

[brb33333-bib-0019] Peng, Z. , Lui, S. S. , Cheung, E. F. , Jin, Z. , Miao, G. , Jing, J. , & Chan, R. C. (2012). Brain structural abnormalities in obsessive‐compulsive disorder: Converging evidence from white matter and grey matter. Asian Journal of Psychiatry, 5(4), 290–296. 23174435 10.1016/j.ajp.2012.07.004

[brb33333-bib-0020] Ping, L. , Su‐Fang, L. , Hai‐Ying, H. , Zhang‐Ye, D. , Jia, L. , Zhi‐Hua, G. , & Zhan‐Jiang, L. (2013). Abnormal spontaneous neural activity in obsessive‐compulsive disorder: A resting‐state functional magnetic resonance imaging study. PLOS ONE, 8(6), e67262. 10.1371/journal.pone.0067262 23826251 PMC3695040

[brb33333-bib-0021] Qiu, L. , Fu, X. , Wang, S. , Tang, Q. , Chen, X. , Cheng, L. , & Tian, L. (2017). Abnormal regional spontaneous neuronal activity associated with symptom severity in treatment‐naive patients with obsessive‐compulsive disorder revealed by resting‐state functional MRI. Neuroscience Letters, 640, 99–104. 28104431 10.1016/j.neulet.2017.01.024

[brb33333-bib-0022] Ruscio, A. M. , Stein, D. J. , Chiu, W. T. , & Kessler, R. C. (2010). The epidemiology of obsessive‐compulsive disorder in the national comorbidity survey replication. Molecular Psychiatry, 15(1), 53–63. 10.1038/mp.2008.94 18725912 PMC2797569

[brb33333-bib-0023] Sachdev, P. S. , McBride, R. , Loo, C. K. , Mitchell, P. B. , Malhi, G. S. , & Croker, V. M. (2001). Right versus left prefrontal transcranial magnetic stimulation for obsessive‐compulsive disorder: A preliminary investigation. Journal of Clinical Psychiatry, 62(12), 981–984. 10.4088/jcp.v62n1211 11780880

[brb33333-bib-0024] Tomasino, B. , & Fabbro, F. (2016). Increases in the right dorsolateral prefrontal cortex and decreases the rostral prefrontal cortex activation after‐8 weeks of focused attention based mindfulness meditation. Brain & Cognition, 102, 46–54. 26720411 10.1016/j.bandc.2015.12.004

[brb33333-bib-0025] Xia, J. , Fan, J. , Du, H. , Liu, W. , Li, S. , Zhu, J. , & Zhu, X. (2019). Abnormal spontaneous neural activity in the medial prefrontal cortex and right superior temporal gyrus correlates with anhedonia severity in obsessive‐compulsive disorder. Journal of Affective Disorders, 259, 47–55. 10.1016/j.jad.2019.08.019 31437701

[brb33333-bib-0026] Xu, T. , Zhao, Q. , Wang, P. , Fan, Q. , Chen, J. , Zhang, H. , & Wang, Z. (2018). Altered resting‐state cerebellar‐cerebral functional connectivity in obsessive‐compulsive disorder. Psychological Medicine, 49, 1156–1165. 10.1017/S0033291718001915 30058519

[brb33333-bib-0027] Yang, X. , Hu, X. , Tang, W. , Li, B. , Yang, Y. , Gong, Q. , & Huang, X. (2019). Multivariate classification of drug‐naive obsessive‐compulsive disorder patients and healthy controls by applying an SVM to resting‐state functional MRI data. BMC Psychiatry, 19(1), 210[Electronic Resource]. 10.1186/s12888-019-2184-6 31277632 PMC6612132

[brb33333-bib-0028] Yang, X. , Luo, J. , Zhong, Z. , Yang, X. , Yao, S. , Wang, P. , & Li, Z. (2019). Abnormal regional homogeneity in patients with obsessive‐compulsive disorder and their unaffected siblings: A resting‐state fMRI study. Front Psychiatry, 10, 452. 10.3389/fpsyt.2019.00452 31316408 PMC6609574

[brb33333-bib-0029] Yang, X. Y. , Sun, J. , Luo, J. , Zhong, Z. X. , Li, P. , Yao, S. M. , & Li, Z. J. (2015). Regional homogeneity of spontaneous brain activity in adult patients with obsessive‐compulsive disorder before and after cognitive behavioural therapy. Journal of Affective Disorders, 188, 243–251. 10.1016/j.jad.2015.07.048 26378734

[brb33333-bib-0030] Ding, Y. , Ou, Y. , Pan, P. , Shan, X. , Chen, J. , Liu, F. , & Guo, W. (2019). Cerebellar structural and functional abnormalities in first‐episode and drug‐naive patients with schizophrenia: A meta‐analysis. Psychiatry Research: Neuroimaging, 283, 24–33. 30500474 10.1016/j.pscychresns.2018.11.009

[brb33333-bib-0031] Zhou, D. D. , Wang, W. , Wang, G. M. , Li, D. Q. , & Kuang, L. (2017). An updated meta‐analysis: Short‐term therapeutic effects of repeated transcranial magnet IC stimulation in treating obsessive‐compulsive disorder. Journal of Affective Disorders, 215, 187–196. 28340445 10.1016/j.jad.2017.03.033

