# Supplementary figures and images for: Reduced functional connectivity of the right dorsolateral prefrontal cortex at rest in obsessive–compulsive disorder
Source: Brain Behav. 2024 Jan 11;14(1):e3333. doi: 10.1002/brb3.3333 (PMC10784187; doi:10.1002/brb3.3333)

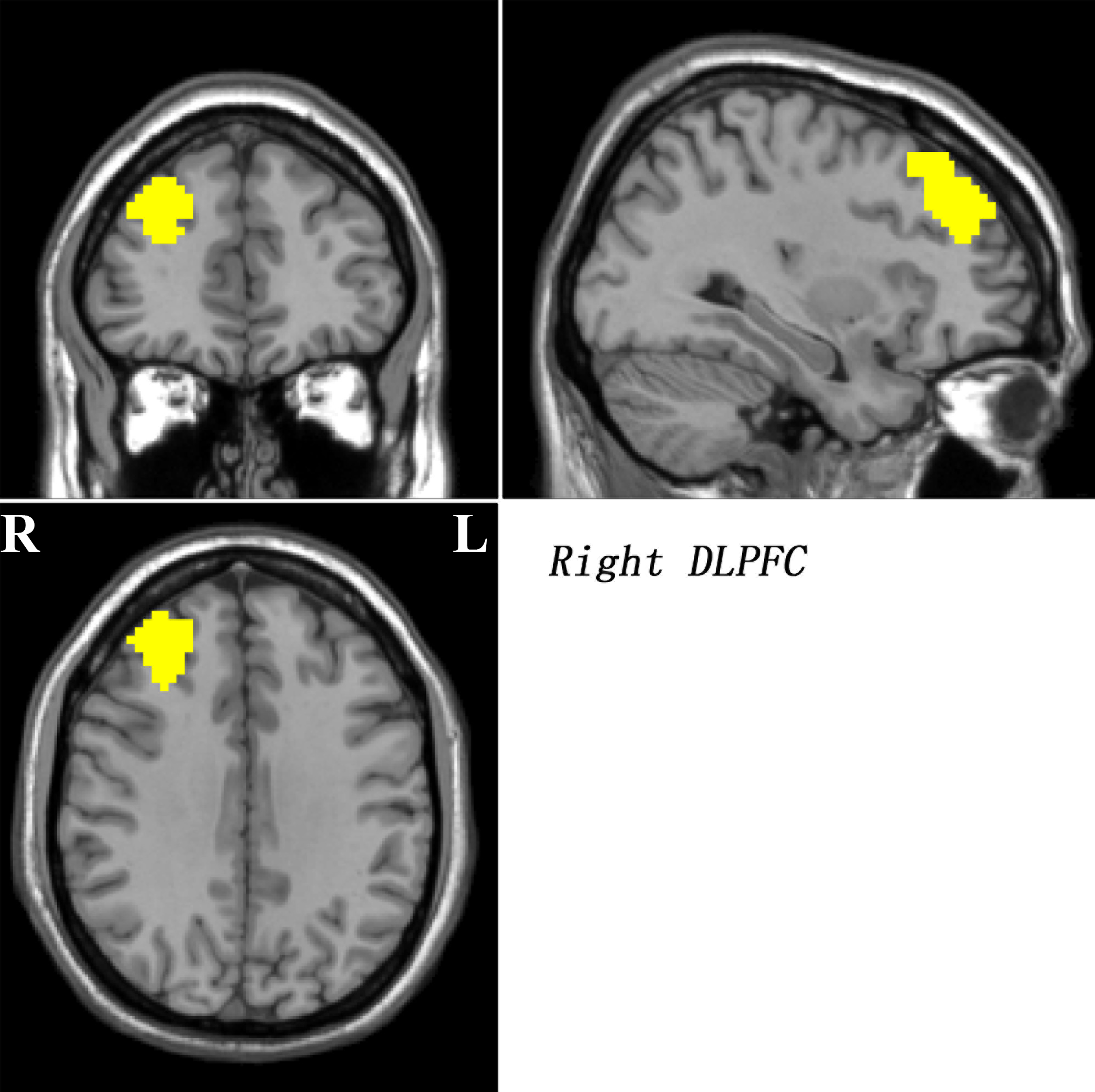

Supplement: Supplementary file 1 — FIGURE S1 Region‐of‐interest mask of the right dorsolateral prefrontal cortex. DLPFC, dorsolateral prefrontal cortex. [file BRB3-14-e3333-s003.tif]

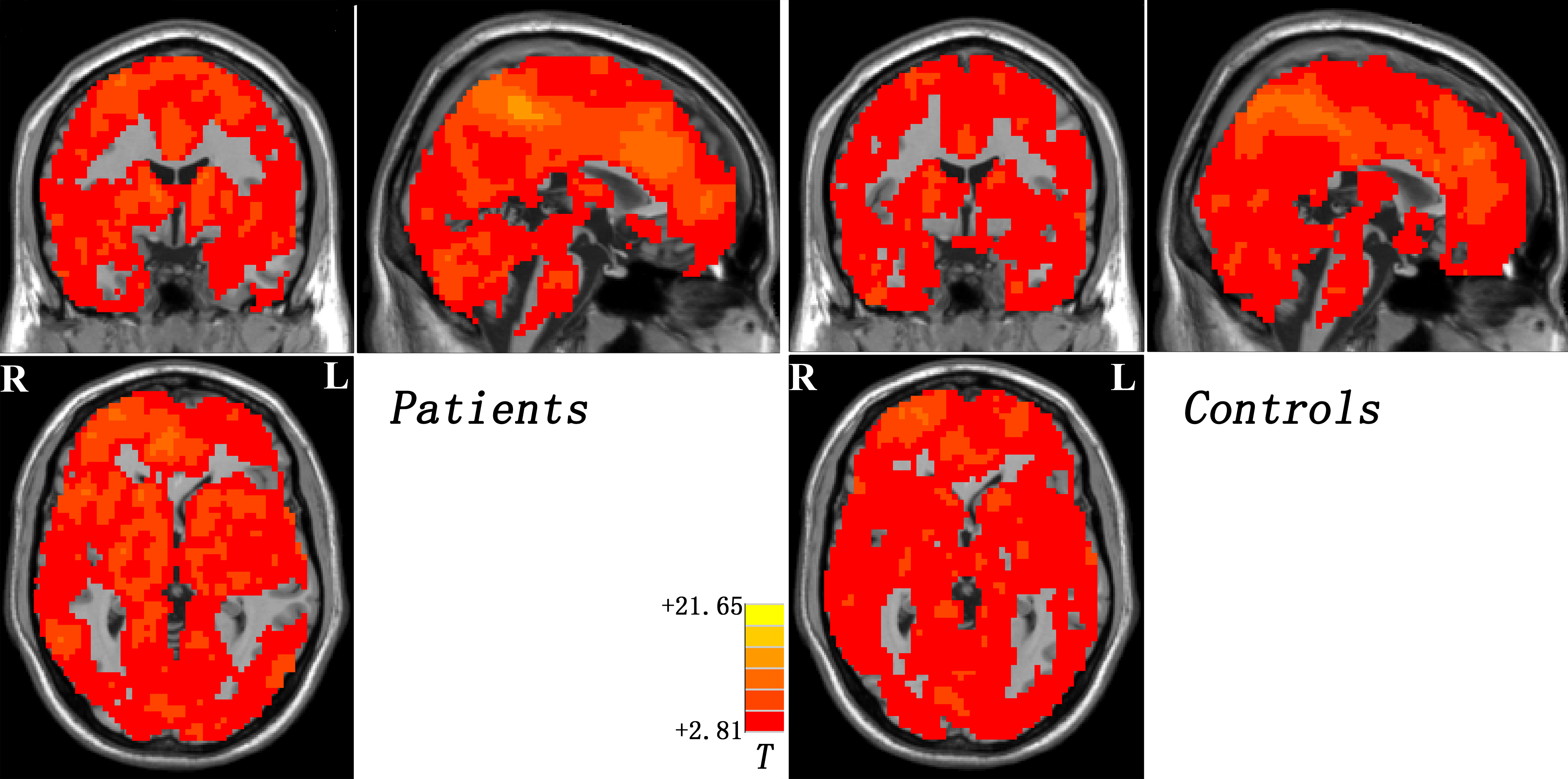

Supplement: Supplementary file 2 — FIGURE S2 Brain regions showing functional connectivities with the right DLPFC within OCD group and HC group. The threshold was set at p < .05 corrected by GRF. Blue denotes reduced FC values in the patients. Color bar indicates the t values from one‐sample t‐tests. L, left side; R, right side; DLPFC, dorsolateral prefrontal cortex; OCD, obsessive–compulsive disorder; HCs, healthy controls. [file BRB3-14-e3333-s001.tif]
