# Supplementary material for: Reduced functional connectivity of the right dorsolateral prefrontal cortex at rest in obsessive–compulsive disorder
Source: Brain Behav. 2024 Jan 11;14(1):e3333. doi: 10.1002/brb3.3333 (PMC10784187; doi:10.1002/brb3.3333)
Supplement: Supplementary file 3 — Supporting Information [file BRB3-14-e3333-s002.docx]

**Materials and methods**

***Image data acquisition and preprocessing***

The following main steps were performed. First, the first 10 volumes were removed. The remaining 230 volumes were collected, and slice timing was corrected. Second, head motion was corrected, and subjects with more than 2 mm of maximal translation and 2° of maximal rotation were excluded. Two HCs were excluded from further analysis due to excessive head motion. Third, the motion corrected functional volumes were spatially normalized to the MNI space and resampled to isotropic voxel size of 3 mm. Fourth, the processed images were smoothed with a 4 mm full-width at half-maximum (FWHM) Gaussian kernel, linearly detrended and band-pass filtered (0.01–0.08 Hz). Fifth, the nuisance covariates, including white matter, 24 head motion parameters, and cerebrospinal fluid time course, were regressed out. Finally, we also scrubbed with a framewise displacement (FD) measure using a threshold of 0.2 together with one preceding and two subsequent volumes (Cunningham, Wernroth, von Knorring, Berglund, & Ekselius, 2011; Han, Chapman, & Krawczyk, 2016; Power, Barnes, Snyder, Schlaggar, & Petersen, 2012).


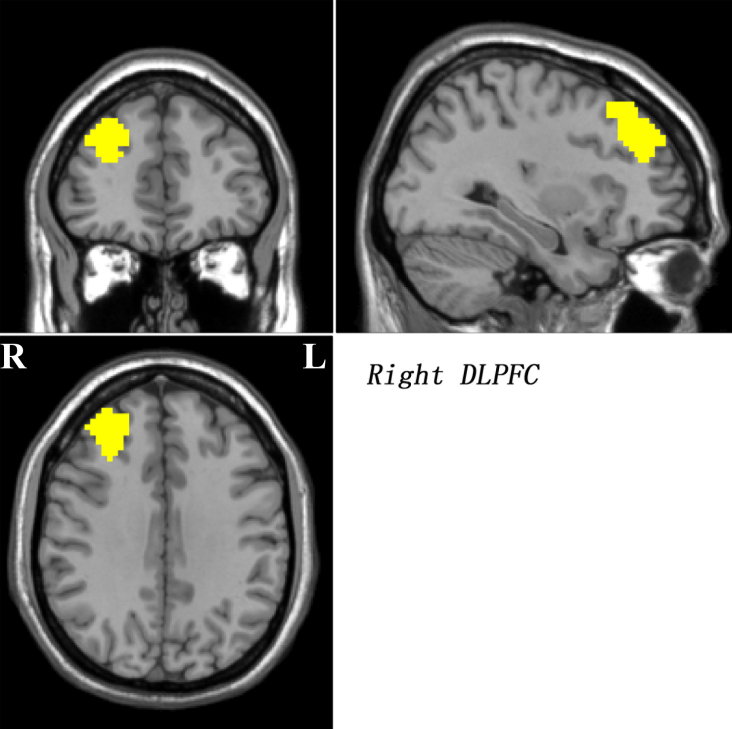


FIGURE S1. Region-of-interest mask of the right dorsolateral prefrontal cortex.

DLPFC = dorsolateral prefrontal cortex


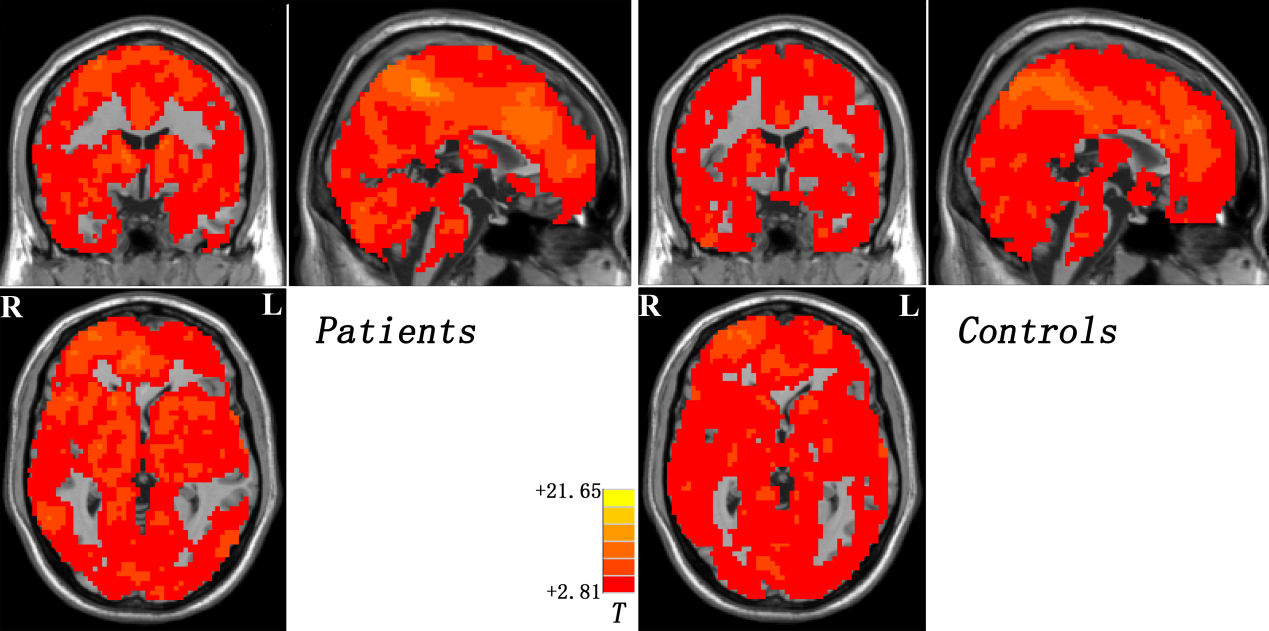


FIGURE S2. Brain regions showing functional connectivities with the right DLPFC within OCD group and HC group. The threshold was set at *p* < 0.05 corrected by GRF. Blue denotes reduced FC values in the patients. Colour bar indicates the *t* values from one-sample *t*-tests. L = left side; R = right side; DLPFC = dorsolateral prefrontal cortex; OCD = obsessive compulsive disorder; HCs = healthy controls.
